# Translation and Cross-Cultural Adaptation of the Toronto Extremity Salvage Score (TESS) for Latin American Spanish–Speaking Patients With Limb Sarcoma: Latin American Spanish TESS Adaptation

**DOI:** 10.1155/2024/7887845

**Published:** 2024-11-06

**Authors:** Oscar Ceballos, Jorge Cabrolier, Begoña Chehade, Francisco Hardoy, Francisco Cortes, Ricardo Tolosa, Orlando Wevar

**Affiliations:** ^1^Division of Orthopaedic Surgery, Dr. Teodoro Gebauer Weisser Orthopaedic Institute, Santiago, Chile; ^2^Division of Orthopedic Surgery, Clinica Santa Maria, Santiago, Chile; ^3^Clinica Alemana, Universidad Del Desarrollo, Santiago, Chile; ^4^Faculty of Medicine, Diego Portales University, Santiago, Chile; ^5^Division of Orthopedic Surgery, Clinica Davila, Santiago, Chile

**Keywords:** outcomes, sarcoma, TESS, translation

## Abstract

**Background and Objectives:** This study aims to translate and culturally adapt the Toronto Extremity Salvage Score (TESS) for Latin American Spanish–speaking patients, enhancing the tool's accessibility for evaluating postsurgical functional outcomes in sarcoma patients across Latin America.

**Methods:** The TESS questionnaires for lower extremity (LE) and upper extremity (UE) were translated and adapted following international guidelines. The process included forward and backward translation, expert committee review, and pretesting with cognitive interviewing. Patients treated for bone or soft tissue tumors in LE or UE were recruited to complete the adapted questionnaires. Test–retest reliability was evaluated by having participants complete the questionnaire again 2 weeks after the initial assessment.

**Results:** A total of 89 participants completed the questionnaires. The study found high internal consistency, with Cronbach's alpha values reaching 0.9437 for LE and 0.9402 for UE. An agreement rate of 98.4% for the global score of TESS-LE (95% confidence interval [CI]: 0.909–1.059) and 93.9% for TESS-UE (95% CI: 0.882–0.995) was observed, demonstrating strong test–retest reliability.

**Conclusions:** The Latin American Spanish version of TESS for both lower and upper extremities is a reliable and culturally appropriate tool for assessing physical function in limb sarcoma patients. Further validation across diverse Latin American populations is encouraged to strengthen its broad applicability.

Summary

• This study adapts the Toronto Extremity Salvage Score (TESS) for Latin American Spanish, demonstrating its reliability for assessing postsurgical outcomes in limb sarcoma patients. High internal consistency and test–retest reliability were confirmed, advocating for broader validation across Latin America.

## 1. Introduction

Sarcomas represent a rare and diverse group of malignant tumors originating from mesenchymal tissues, predominantly affecting the limbs and leading to substantial morbidity. The primary treatment objective for these conditions is limb salvage through surgical resection, utilizing a range of techniques including arthroplasty, allografts, and meticulous vascular and nerve management. Depending on the specific subtype of sarcoma, this may be complemented by chemotherapy, radiotherapy, or both.

Evaluating postsurgical functional outcomes is crucial for determining the success of these interventions, particularly in light of increasing long-term survival rates among patients [1]. The TESS [2] is a globally acknowledged scale extensively used to assess physical disability and health-related quality of life in patients who have undergone limb salvage procedures for sarcoma.

The TESS has seen global adoption and has been transculturally adapted to various languages, including Portuguese [3], Turkish [4], Danish [5], Korean [6], Japanese [7, 8], Chinese [9], Dutch [10, 11], Finnish [12], Italian [13], Greek [14], and German [15]. Despite its extensive utilization, a Spanish version of the TESS, specifically tailored to the Latin American context, is not yet available. Given that over 450 million native Spanish speakers reside in Latin America, creating a culturally appropriate adaptation of the TESS is imperative. This task goes beyond mere translation; it requires thoughtful consideration of the cultural and contextual nuances that could influence patient responses. Ensuring the TESS's reliability in the Spanish-speaking world is essential for healthcare professionals aiming to conduct accurate and effective assessments of patients' quality of life, fostering patient-centered and tailored medical care for this unique population. Consequently, this study is dedicated to adapting the TESS to Latin American Spanish, aiming to provide healthcare professionals with a reliable and practical tool for evaluating the functional outcomes of sarcoma patients in the region.

## 2. Materials and Methods

The research protocol for our study received approval from the Ethics Committee of the Western Metropolitan Health Service in Santiago, Chile, ensuring that all procedures and practices adhere to the highest standards of ethical conduct (approval year: 2022).

The process of translation and adaptation was based on the guidelines described by Beaton et al. [16]. The TESS surveys for the LE and UE were treated distinctly during the process. The initial translation of the English TESS to Latin American Spanish was undertaken by three bilingual experts, with Latin American Spanish as their primary language. This resulted in the preliminary Latin American Spanish–unified version. The translation process was straightforward, with only minor challenges encountered. Specifically, there were only a few phrases that did not make sense when translated to Latin American Spanish, which required the consensus of the expert committee to select culturally relevant alternatives. These minor issues were resolved through iterative revisions to ensure clarity and cultural appropriateness. Subsequently, two bilingual certified translators without medical expertise translated this Latin American Spanish version back into English. The expert committee, comprising authors, two translators, and an invited sarcoma surgeon whose mother tongue is English, reviewed all versions and components of the original questionnaire and the translations, reaching a consensus on the final wording for the Latin American Spanish version of the TESS- LE and UE (Supporting Information 1 and 2).

Consecutive eligible patients who visited the outpatient clinic between March and September 2023 for follow-up after previous surgery for bone or soft tissue tumors of the extremities were approached to fill out the translated and adapted TESS. To qualify, patients needed to (i) be aged 18 years or older, (ii) have at least 6 months since their surgical treatment for an aggressive benign or malignant bone tumor or soft tissue sarcoma, and (iii) show no evidence of local or systemic recurrent disease. Patients with communication impairments or those unable to complete the questionnaires independently were excluded from the survey. Data gathered on the participating patients included age, gender, diagnosis, and tumor location.

Eligible patients were approached to join the study by an orthopedic surgery resident during their visit to the outpatient clinic. The surveys were made available through an online platform, accessible via a link sent to participants through email or text message, allowing them to respond using their smartphones [17]. Participants were instructed to complete the first questionnaire while waiting for their appointment. Two weeks following the completion of the initial questionnaire, a second survey was distributed, with patients being prompted to respond as promptly as possible. A 2-week interval was selected for the retest to minimize recall bias. Responses were automatically recorded and stored. To facilitate the test–retest analysis, each set of questionnaires was correlated using a unique code.

Cronbach's alpha was employed to determine the internal consistency of the instrument, providing insight into how well the items within the questionnaire measure the same construct. To evaluate the stability of the instrument over time and assess test–retest reliability, Cohen's kappa coefficient was calculated. This involved comparing responses from the first and second administrations of the questionnaire for each individual item and for the total score. A higher kappa value indicates greater reliability and consistency in the responses across the two time points.

The total score for every questionnaire was calculated. In order to analyze the agreement of the total test–retest scores, the Bland–Altman method was applied, estimated through a simple linear regression with the elimination of the constant term. A significance level of 5% was employed, confidence intervals were set at 95%, and data processing was conducted using STATA version 18.0.

## 3. Results

During the translation and cross-cultural adaptation phase of the TESS-LE and TESS-UE questionnaires, the translators and expert committee successfully navigated the process without encountering significant linguistic or cultural hurdles. This meticulous work culminated in the creation of the Latin American Spanish versions of the TESS-LE and TESS-UE, which can be found as Supporting Information.

Out of the 95 patients who filled out the questionnaires, six were excluded due to incomplete data. Consequently, 89 participants (52% female), with a mean age of 43 years (ranging from 18 to 83 years) were included in the final analysis. A comprehensive breakdown of the patients' characteristics is provided in Table 1.

**Figure 1:**
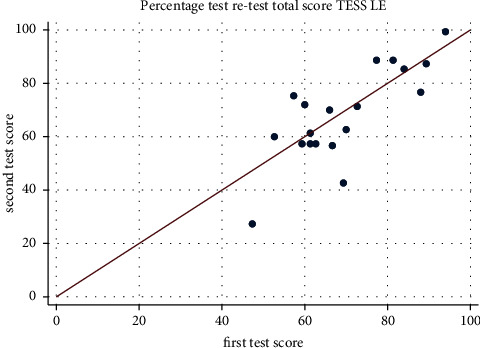
The chart illustrates the test–retest reliability of the Latin American Spanish TESS-LE. The line on the plot represents the perfect correlation between the test administrations.

**Figure 2:**
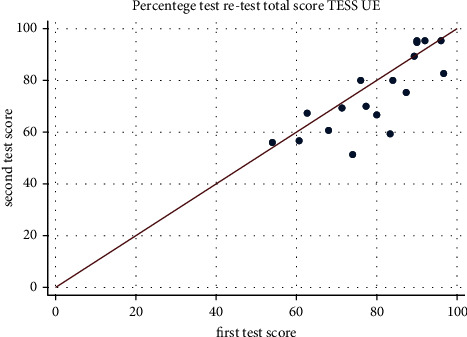
The chart illustrates the test–retest reliability of the Latin American Spanish TESS-UE. The line on the plot represents the perfect correlation between the test administrations.

The TESS-LE and TESS-UE both exhibited excellent internal consistency, with Cronbach's alpha values of 0.9437 and 0.9402, respectively. Regarding retest reliability, 36% of LE patients and 84% of UE patients participated in the retest phase. Each item, when individually evaluated using Cohen's kappa coefficient, showed significant concordance. The Bland–Altman method indicated a 98.4% agreement for the global score of TESS-LE (95% confidence interval [CI]: 0.909–1.059), as shown in Figure 1, and a 93.9% agreement for TESS-UE (95% CI: 0.882–0.995), as illustrated in Figure 2, confirming strong test–retest reliability for both assessments.

## 4. Discussion

Sarcoma research is gradually gaining traction in Latin America, although it is still in its nascent stages. The TESS is a prevalent tool among researchers in Chile and various Latin American countries for evaluating physical function in limb sarcoma patients. Despite its wide use, there was a conspicuous absence of a validated Latin American Spanish version of the tool, a gap that our study sought to fill. By translating and culturally adapting the TESS for both LE and UE, we have provided a tool that is linguistically and culturally tailored to the Latin American context.

The cultural adaptation process from the Canadian to the Latin American version of the TESS was minimal. Despite the geographic and linguistic differences, there are shared elements in the way individuals in these regions approach their routine tasks and engage in physical activities. This facilitated a smoother translation and adaptation process, requiring only slight modifications to ensure that the questionnaire resonated with and was relevant to the Latin American population.

A total of six questionnaires were excluded from the analysis due to incomplete information. Four patients had left some questions unanswered, and the ambiguity surrounding whether this meant the item was “not applicable” or simply overlooked prompted the authors to exclude these entries to preserve data integrity. In addition, two patients marked an unusually high number of questions as “not applicable” (more than 20). Despite the potential to calculate a score, concerns regarding the reliability of these responses led the authors to exclude these questionnaires.

The Latin American versions of the TESS-LE and TESS-UE demonstrated internal consistencies and test–retest reliabilities that were on par with the original TESS version, as well as other translated and validated versions of the tool. This indicates that the adaptations made for the Latin American context have successfully preserved the reliability and consistency of the TESS, ensuring its effectiveness in evaluating the functional outcomes of patients with limb sarcomas in this region.

Contrary to the Dutch version of TESS [10, 11], which reported a noticeable ceiling effect of 39.6% for the UE questionnaire, the Latin American Spanish version displayed a distinct profile. Only 6.45% of patients achieved the maximum score for the UE questionnaire, and none did so for the LE questionnaire. This underscores the tool's capacity for precise discrimination across varying degrees of functionality, maintaining its accuracy even in cases of high performance.

Although the sample size was larger in the LE group, reflecting the higher prevalence of LE musculoskeletal tumors compared to UE tumors, the return rates for the second questionnaire varied significantly. Only 36% of the LE group participants completed the second questionnaire, compared to 83% of the UE group. This discrepancy resulted in a smaller sample size for assessing test–retest validity within the LE group. While the exact reasons for the lower return rate in the LE group are unclear, several factors could contribute to this issue, such as differences in patient motivation or challenges related to completing the questionnaire at home. To improve response rates in future studies, strategies such as follow-up reminders, simplifying the survey process, or providing additional incentives could be considered.

Potential limitations of this study include the relatively small sample size and the possibility of selection bias, as participants were recruited from a single institution. In addition, the cultural diversity within Latin America means that further validation in different countries and regions is necessary to ensure the tool's broad applicability. Future research should focus on expanding the sample size and including diverse populations from various Latin American countries to confirm the reliability and validity of the adapted TESS.

## 5. Conclusion

The Latin American Spanish version of the TESS questionnaire for UE and LE stands as a reliable tool for assessing patient-reported physical function in individuals undergoing limb salvage surgery for both benign and malignant bone and soft tissue tumors. This adaptation ensures linguistic and cultural relevance, enhancing the tool's applicability within Latin American populations. Moreover, the availability of the Latin American Spanish TESS paves the way for its inclusion in future international cross-cultural studies within the field of orthopedic oncology, fostering a more inclusive and comprehensive global research environment. While our findings affirm the reliability of the Latin American Spanish TESS, further research is encouraged to validate the adapted TESS in broader Latin American populations, considering the cultural differences within the region that might affect the applicability of the score. This will help ensure that the tool can be reliably used across diverse cultural contexts within Latin America.

## Figures and Tables

**Table 1:** Patient and tumor characteristics of individuals who completed the TESS questionnaire.

*N*	TESS-LE	TESS-UE
	57	32
Age: mean (range)	43 (18–83)	41.3 (22–79)
Gender: % male	47.4%	48.4%

*Location n (%)*		
Shoulder	0	1 (3)
Humerus	0	11 (34)
Upper arm (soft tissue)	0	7 (22)
Radius	0	6 (19)
Metacarpals	0	1 (3)
Digits	0	5 (16)
Femur	32 (56)	0
Leg (soft tissue)	7 (12)	0
Knee	5 (9)	0
Tibia	8 (14)	0
Fibula	2 (4)	0
Foot	2 (4)	0
Missing data∗	1 (2)	1 (3)

*Primary tumor n (%)*		
Atypical cartilaginous tumor	6 (11)	6 (19)
Chondrosarcoma grade 2/3	5 (9)	2 (6)
Osteosarcoma	23 (40)	8 (25)
Soft tissue sarcoma	8 (14)	6 (19)
Diffuse tenosynovial giant cell tumor	5 (9)	2 (6)
Cartilaginous tumor, benign	1 (2)	0
Bone other, malignant	2 (4)	1 (3)
Soft tissue other, benign	0	1 (3)
Bone other, benign	6 (11)	5 (16)
Missing data∗	1 (2)	1(3)

^∗^Baseline characteristics were unavailable for two patients (1 LE and 1 UE) due to recording issues.

## Data Availability

The data that support the findings of this study are available from the corresponding author upon reasonable request.

## Supporting Information

Copies of the translated TESS questionnaires for both lower extremity and upper extremity are provided as the supporting information.
