# Supplementary material for: Translation and Cross-Cultural Adaptation of the Toronto Extremity Salvage Score (TESS) for Latin American Spanish–Speaking Patients With Limb Sarcoma: Latin American Spanish TESS Adaptation
Source: Int J Surg Oncol. 2024 Nov 6;2024:7887845. doi: 10.1155/2024/7887845 (PMC11561176; doi:10.1155/2024/7887845)
Supplement: Supporting Information — Additional supporting information can be found online in the Supporting Information section. [file 7887845.f1.zip › TESS_LE_Spanish.pdf]

TESS Extremidad Inferior

		Imposible de realizar	Extremadamente dificil	Moderadamente dificil	Un poco dificil	Nada dificil	Esta tarea no es aplicable a mi
1	Ponerse pantalones es:	1	2	3	4	5	0
2	Ponerse zapatos es:	1	2	3	4	5	0
3	Ponerse un par de calcetines o medias es:	1	2	3	4	5	0
4	Ducharse es:	1	2	3	4	5	0
5	Las tareas domésticas ligeras, como ordenar y quitar el polvo, son:	1	2	3	4	5	0
6	La jardinería es:	1	2	3	4	5	0
7	Preparar las comidas es:	1	2	3	4	5	0
8	Ir de compras es:	1	2	3	4	5	0
9	Las tareas domesticas pesadas, como pasar la aspiradora y mover los muebles, son:	1	2	3	4	5	0
10	Entrar y salir de la bañera es:	1	2	3	4	5	0
11	Levantarse de la cama:	1	2	3	4	5	0
12	Levantarse de una silla es:	1	2	3	4	5	0
13	Arrodillarse es:	1	2	3	4	5	0
14	Inclinarse para recoger algo del suelo es:	1	2	3	4	5	0
15	Subir una escalera es:	1	2	3	4	5	0

16	Bajar una escalera es:	1	2	3	4	5	0
17	Conducir un automovil es:	1	2	3	4	5	0
18	Caminar dentro de la casa es:	1	2	3	4	5	0
19	Caminar en el exterior es:	1	2	3	4	5	0
20	Sentarse es:	1	2	3	4	5	0
21	Subir o bajar colinas o una rampa es:	1	2	3	4	5	0
22	Estar de pie es:	1	2	3	4	5	0
23	Levantarse desde estar arrodillado:	1	2	3	4	5	0
24	Entrar y salir de un automovil es:	1	2	3	4	5	0
25	Participar en actividades sexuales es:	1	2	3	4	5	0
26	Cumplir con mis deberes habituales en el trabajo es: (el trabajo incluye un trabajo fuera de casa o como dueña/o de casa).	1	2	3	4	5	0
27	Trabajar el número de horas que trabajo habitualmente es: (trabajar incluye tanto el trabajo fuera de casa como el de dueña/o de casa).	1	2	3	4	5	0
28	Participar en mis actividades de ocio habituales es:	1	2	3	4	5	0
29	Socializar con los amigos y la familia es:	1	2	3	4	5	0
30	Participar en mis actividades deportivas habituales es:	1	2	3	4	5	0
