# Supplementary material for: Translation and Cross-Cultural Adaptation of the Toronto Extremity Salvage Score (TESS) for Latin American Spanish–Speaking Patients With Limb Sarcoma: Latin American Spanish TESS Adaptation
Source: Int J Surg Oncol. 2024 Nov 6;2024:7887845. doi: 10.1155/2024/7887845 (PMC11561176; doi:10.1155/2024/7887845)
Supplement: Supporting Information — Additional supporting information can be found online in the Supporting Information section. [file 7887845.f1.zip › TESS_UE_Spanish.pdf]

TESS Extremidad Superior

		Imposible de realizar	Extremadamente dificil	Moderadamente dificil	Un poco dificil	Nada dificil	Esta tarea no es aplicable a mi
**1**	**Ponerse pantalones es:**	1	2	3	4	5	0
**2**	**Amarrar los cordones de los zapatos es:**	1	2	3	4	5	0
**3**	**Ponerse un par de calcetines o medias es:**	1	2	3	4	5	0
**4**	**Ducharse es:**	1	2	3	4	5	0
**5**	**Vestir mis brazos y la parte superior del cuerpo es:**	1	2	3	4	5	0
**6**	**Abotonar una caminsa es:**	1	2	3	4	5	0
**7**	**Anudar una corbata o un pañuelo en el cuello de una camisa es:**	1	2	3	4	5	0
**8**	**Maquillarse o afeitarse es:**	1	2	3	4	5	0
**9**	**Lavarse los dientes es:**	1	2	3	4	5	0
**10**	**Peinarse es:**	1	2	3	4	5	0
**11**	**Hacer tareas domésticas ligeras es:**	1	2	3	4	5	0
**12**	**La jardinería es:**	1	2	3	4	5	0
**13**	**Preparar y servir comidas es:**	1	2	3	4	5	0
**14**	**Cortar la comida mientras come es:**	1	2	3	4	5	0

**15**	**Beber de un vaso es:**	1	2	3	4	5	0
**16**	**Realizar tareas domésticas pesadas es:**	1	2	3	4	5	0
**17**	**Ir de compras es:**	1	2	3	4	5	0
**18**	**Dar o recibir vuelto (es decir, monedas o billetes) es:**	1	2	3	4	5	0
**19**	**Llevar una bolsa de la compra o un maletín es:**	1	2	3	4	5	0
**20**	**Levantar una caja a un estante sobre el nivel de su cabeza es:**	1	2	3	4	5	0
**21**	**Girar una llave en la cerradura es:**	1	2	3	4	5	0
**22**	**Empujar o tirar para abrir una puerta es:**	1	2	3	4	5	0
**23**	**Escribir es:**	1	2	3	4	5	0
**24**	**Recoger objetos pequeños es:**	1	2	3	4	5	0
**25**	**Cumplir con mis deberes habituales en el trabajo es: (el trabajo incluye un trabajo fuera de casa o como dueña/o de casa).**	1	2	3	4	5	0
**26**	**Trabajar el número de horas que trabajo habitualmente es: (trabajar incluye tanto el trabajo fuera de casa como el de dueña/o de casa).**	1	2	3	4	5	0
**27**	**Participar en mis actividades de ocio habituales es:**	1	2	3	4	5	0
**28**	**Socializar con los amigos y la familia es:**	1	2	3	4	5	0
**29**	**Participar en mis actividades deportivas habituales es:**	1	2	3	4	5	0
